# Nanoscale Changes on RBC Membrane Induced by Storage and Ionizing Radiation: A Mini-Review

**DOI:** 10.3389/fphys.2021.669455

**Published:** 2021-06-04

**Authors:** Andrea M. López-Canizales, Aracely Angulo-Molina, Adriana Garibay-Escobar, Erika Silva-Campa, Miguel A. Mendez-Rojas, Karla Santacruz-Gómez, Mónica Acosta-Elías, Beatriz Castañeda-Medina, Diego Soto-Puebla, Osiris Álvarez-Bajo, Alexel Burgara-Estrella, Martín Pedroza-Montero

**Affiliations:** ^1^Departamento de Ciencias Químico-Biológicas, Universidad de Sonora, Hermosillo, Mexico; ^2^Departamento de Investigación en Física, Universidad de Sonora, Hermosillo, Mexico; ^3^Departamento de Ciencias Químico-Biológicas, Universidad de las Américas, Puebla, Mexico; ^4^Departamento de Física, Universidad de Sonora, Hermosillo, Mexico

**Keywords:** RBC membrane, nanoalterations, ionizing radiation, blood storage, confocal microscopy, Raman, scanning electron microscopy, atomic force microscopy

## Abstract

The storage lesions and the irradiation of blood cellular components for medical procedures in blood banks are events that may induce nanochanges in the membrane of red blood cells (RBCs). Alterations, such as the formation of pores and vesicles, reduce flexibility and compromise the overall erythrocyte integrity. This review discusses the alterations on erythrocytic lipid membrane bilayer through their characterization by confocal scanning microscopy, Raman, scanning electron microscopy, and atomic force microscopy techniques. The interrelated experimental results may address and shed light on the correlation of biomechanical and biochemical transformations induced in the membrane and cytoskeleton of stored and gamma-irradiated RBC. To highlight the main advantages of combining these experimental techniques simultaneously or sequentially, we discuss how those outcomes observed at micro- and nanoscale cell levels are useful as biomarkers of cell aging and storage damage.

## Introduction

Irradiation of blood components is the only accepted procedure for preventing transfusion-associated graft-versus-host disease (TA-GVHD; Santacruz-Gomez et al., 2016). The standard dose is 25 Gy of gamma rays or X-rays (Manduzio, 2018). The effects of ionizing radiation on biochemical properties of red blood cell (RBC) membrane are well-documented and disclosing that lymphocyte apoptosis is responsible for lymphocytopenia in the early stage after irradiation (Sellins and Cohen, 1987; Moroff and Luban, 1994; Moroff and Luban, 1997; Cui et al., 1999). Radiation acts negatively on the mitotic activity of lymphocytes responsible for triggering TA-GVHD, rendering them unviable (Xu et al., 2012). The radiation accentuates the effects of storage, promoting the generation of reactive oxygen species, membrane lipid peroxidation, and increased permeability of RBC’s membrane to potassium, sodium, hemoglobin (Hb), and lactate dehydrogenase, and a decrease in RBC’s deformability (Ramirez et al., 1987; Anand et al., 1997; Hirayama et al., 2005; Zbikowska et al., 2014; Acosta-Elías et al., 2017; Adams et al., 2018).

In blood banks, RBC undergoes time-dependent biochemical changes collectively known as storage lesions (Antonelou and Seghatchian, 2016; Kozlova et al., 2017). During storage, it is common to observe reductions of internal pH, depletion of adenosine triphosphate (ATP), 2,3-diphosphoglycerate (2,3-DPG), nitric oxide (NO), and glucose, as well as the loss of activity of glutathione and glutathione peroxidase. Conversely, there is an increase in the levels of free Hb, potassium, lactic acid, and calcium (D’Alessandro et al., 2015; Kim et al., 2015; D’Alessandro et al., 2017; Barshtein et al., 2018; Yoshida et al., 2019). These changes produce structural modifications at the nanoscale level, such as pores and vesicle formation, compromising essential functional and structural properties of RBC (Santacruz-Gomez et al., 2014; D’Alessandro et al., 2015; Acosta-Elías et al., 2017; Koch et al., 2019).

The depletion of ATP impacts the membrane elasticity, intracellular viscosity, and the optimal surface area/volume ratio (Kim et al., 2015). Over time, the morphology changes from normal biconcave disc cells to irreversibly deformed spherocytes (Bosman et al., 2008; Koch et al., 2019), mainly due to the membrane’s shedding of vesicles. These vesicles are generally produced during maturation to remove damaged cell components, preventing their removal from circulation by the spleen (Huisjes et al., 2018). Vesiculation is a cellular process that may trigger intracellular communication. The RBCs do not undergo classical apoptosis. Instead, they can be removed from the circulation by cell death due to hemolysis (osmotic shock) or through the antagonistic effects of phosphatidylserine (PS) and CD47 on the phagocytic activity of macrophages, present in the spleen and liver. Recently, RBCs are not considered inert bystanders but active agents in intercellular signaling, immune function, and inflammatory processes (Arias and Arias, 2017). In this sense, the RBC vesiculation process promotes cell-cell communication. They function to store and transport proteins and lipids, activate cytokine cascades, growth factors, and even activate signaling pathways in target cells (Alaarg et al., 2013; Huang-Doran et al., 2017; Karsten and Herbert, 2019; Zhang et al., 2019). The vesicles are produced by the dislocation of the phospholipids of the plasmatic membrane and cytoskeleton involving protein oxidation (Sut et al., 2017). Along the life span of RBCs, their surface area decreases by approximately 20% due to vesicle release (Werre et al., 2004). This decrease in size is used as a marker for aging and storage lesions (Girasole et al., 2010; Dinarelli et al., 2018).

On the other hand, albumin is the most abundant plasma protein. When albumin concentration falls (in low circulation flow *in vitro*; Jay, 1975; Talstad et al., 1983; Reinhart and Nagy, 1995; Reinhart et al., 2015), RBCs suffer reversible alterations until transfusion, like echinocytic morphology transformation, increasing aggregation, sedimentation, and viscosity. These phenomena occur since this protein is responsible for eliminating substances from the outer half of the lipid bilayer of the RBC, for example, lysophosphatidylcholine, which accumulates during metabolic depletion.

Some biochemical values already mentioned are currently used in the blood bank as quality markers in blood cells, such as potassium leakage manifested by changes in cations concentration due to falling ATP levels, or hemolysis, a phenomenon occurring in a more rigid membrane, which is susceptible to rupture (Wallas, 1979; Antwi-Baffour et al., 2019). Nanoscale analysis can be a handy tool to determine biochemical and structural changes, such as alterations in protein domains of the erythrocyte membrane caused by storage injury and irradiation. These nanoalterations cannot be detected earlier through current blood bank techniques until the damage is clearly present in the RBC (Zheng and Li, 2012).

The atomic force microscopy (AFM) technique allows studying the erythrocyte membrane’s roughness, considered as a parameter proportional to the cell deformability (Santacruz-Gomez et al., 2014). The main disadvantage of AFM is that it is possible to confuse two nanostructures that look the same physical but have different biochemical compositions. On the other hand, Raman spectroscopy can detect the biochemical fingerprint of micro-conformational changes associated with membrane proteins and lipids and Hb’s oxygenation states in erythrocytes (Li et al., 2012). In this sense, the use of techniques, such as AFM and Raman, allows the detection of potential early alterations to make better decisions when administering the patient’s blood bag. This information would help broaden the panorama toward an early medical diagnosis and, in the future, could adapt these techniques to the blood bank for routine analysis (Dulińska et al., 2006).

Understanding both the micro- and nanobiochemical alterations that preclude the healthy function of RBC is a challenging task with huge pharmaceutical and medical implications. This mini-review explores a set of experimental biophysics and microscopy techniques that may address and shed light on the correlation of biomechanical and biochemical transformations induced in stored and gamma-irradiated RBCs.

## Storage-Induced Alterations in RBC and Their Characterization

Among the changes induced by blood bank storing, RBC’s vesiculation is probably the result of spontaneous curvature, compression, and altered membrane protein conformation (Li and Lykotrafitis, 2015). Disturbances in the interaction between the cytoskeleton and the lipid bilayer may lead to microparticle detachment (Leal et al., 2018; Freitas Leal et al., 2020). This cooperative phenomenon affects the structure and conformation of spectrin and actin proteins, located in the cytoskeleton, the two domain structures of band 3, one of them present in the cytosol and the other in the lipid bilayer and the ankyrin-binding protein (Kim et al., 2015; Lux, 2016; Dinarelli et al., 2018). The formation of band 3-IgG complexes and phosphatidylserine exposure can also promote vesiculation (Girasole et al., 2010; Huisjes et al., 2018). Thus, clustered band 3 induces autologous IgG binding, and the impaired interaction of band 3 to ankyrin/spectrin network induces the shedding of vesicles containing the clustered band 3 and prevents IgG opsonization and RBC clearance. Therefore, vesiculation is suggested as an RBC mechanism to avoid cell elimination from circulation. Hence, vesicles are removed by recognition of RBC “eat me” signals, such as PS and specific band 3 cleavage products that react with senescent antigens (Alaarg et al., 2013). The composition of the microparticles may vary depending on the duration and storage conditions (Piccin et al., 2015).

Micro- and nanoscale characterization techniques have been used to elucidate the processes that deteriorate RBC functionality (Chasis and Mohandas, 1986; Girasole et al., 2001, 2010; Huang et al., 2011; Bhaduri et al., 2014; Santacruz-Gomez et al., 2014; Mukherjee et al., 2015; Acosta-Elías et al., 2017; Rico et al., 2018; Depond et al., 2020; Lima et al., 2020).

For surface studies, the laser confocal scanning microscopy (LCSM) is the most commonly used technique, as it allows the localization of a specific molecule in the cell and organelles (Collazo et al., 2005), but its resolution cannot reach the nanometric scale for alterations. However, this simple technique facilitates the understanding of the dynamics of proteins and lipids in the RBCs membrane, e.g., the bicarbonate/chloride exchanger band 3, cytoskeleton proteins, such as spectrin, ankyrin, and actin, 4.1 protein, glycophorin C, and even Hb and ATP (Campanella et al., 2005; Antonelou et al., 2010; Chu et al., 2012). Even more, LCSM for immunostaining protocols (Antonelou et al., 2010) disclosed how the distribution of erythrocyte proteins band 3, spectrin, and Hb is affected after 42 days of storage. Also, different oxidative effects on RBC can be analyzed using LCSM, such as alterations on the band 3 protein and spectrin network that can cause changes in biconcave morphology (e.g., equinocitic, stomatocytic, or spherocitic shape) and may provoke the loss of cell functionality (deformability and elasticity; Oore-ofe et al., 2017). A deeper study of these conformational changes of membrane proteins and lipids, as well as the oxygenation states of Hb in erythrocytes, can be made using Raman spectroscopy (RS; Asghari-Khiavi et al., 2010; Chu et al., 2012; Parshina et al., 2013; Acosta-Elías et al., 2017). In this way, RS is a precise technique that can provide information about vibrational states as chemical composition, molecular structure, and molecular interactions in cells and tissues (Choo-Smith et al., 2002; Parshina et al., 2013). It can follow the evolution of biological events in real time, but sometimes the fluorescence signal from water or related aqueous substances may hinder the Raman signal. This analytical tool has been widely used to study blood and its components, ranging from basic research on hemoglobin oxygenation to forensic investigations (Kang et al., 2008; Huang et al., 2011; Santacruz-Gomez et al., 2014; Buckley et al., 2016; Acosta-Elías et al., 2017; Atkins et al., 2017; Gautam et al., 2018; Burgara-Estrella et al., 2020). In particular, the oxygenated and deoxygenated Raman resonant hemoglobin modes have been used as indicators to describe RBC’s integrity (Wood et al., 2001). In a particular case of Raman setup called *resonant* Raman, we can select the modes to measure only isolated Hb changes. For a given resonant wavelength, the response generated exceeds the signal from the membrane’s proteins (Acosta-Elías et al., 2017). Besides, resonant Raman can track not only the structure of Hb during storage but also the fluidity of the RBC membrane changes during cell aging.

Additionally, scanning electron microscopy (SEM) allows us to visualize the progressive changes in RBC morphology related to storage injury (Usry et al., 1975; Berezina et al., 2002; Mustafa et al., 2016). SEM techniques provide us with an excellent image of the topology of the RBC membrane surface with the capacity to perform an elemental analysis (through EDS) of selected regions. The main drawback of this technique is the complicated preparation of biological samples, generating the alteration of them in many cases. Among the changes observed by SEM, the most significant are the alterations in the typical biconcave RBC conformation, making them stiffer and prone to breakage with diminished resistance to hemolysis (Girasole et al., 2007; Kor et al., 2009; Acosta-Elías et al., 2017). In this line, the SEM recorded changes during storage, including the loss of RBC’s discoid shape and their transformation to spherocytes because of the decrease in the surface/volume ratio following the vesiculation process (Geekiyanage et al., 2019). These phenomena are successfully observed after 14 days with nanoalterations by day 21 in pores and high free Hb (Mustafa et al., 2016; Acosta-Elías et al., 2017). Despite these remarkable results, preparation of biological (non-conductive) samples in SEM involves a gold coating that complicates to see fast changes in RBC structure and preclude following in short times their evolution.

The rapid assessment of membrane nanomodifications in RBCs may be accomplished using AFM (Girasole et al., 2001, 2007; Asghari-Khiavi et al., 2010; Santacruz-Gomez et al., 2014; Marzec et al., 2015; Acosta-Elías et al., 2017; Kozlova et al., 2018; Spyratou et al., 2019; Burgara-Estrella et al., 2020). This technique allows the tracking in short times with minimal sample preparation, during the blood storage, the progressive impairment of the spectrin tetramers network’s dynamical properties connected to actin complexes, and the bilayer’s integral proteins (Moroz et al., 2010; Dinarelli et al., 2018; Kozlova et al., 2018, 2019). Here, the biochemical changes render a diminishing of roughness membrane. This fact is noteworthy because roughness is considered a cell health parameter proportional to its deformability. The storage process induces the appearance of nanopores and nanovesicles that modify the microstructure and deformability of RBC and, therefore, their function (Girasole et al., 2007, 2010; Santacruz-Gomez et al., 2014; Acosta-Elías et al., 2017; Kozlova et al., 2019). Consequently, the storage process triggers a deterioration in the functionality of RBC, mainly in the oxidative metabolism at the level of protein oxidation, including hemoglobin, lipid oxidation, and metabolic alterations, such as lactate accumulation 2,3-DPG depletion. Stored erythrocytes may cause adverse effects on the circulatory cycle, non-transferrin bound iron, insufficient nitric oxide bioavailability, as well as altered infusion and damage to immune modulation (e.g., inflammation), and also cardiac electrophysiological alterations (Yoshida et al., 2019; Reilly et al., 2020).

## RBC Alterations by Ionizing Radiation and Characterization Techniques

Irradiation of blood is recommended to prevent TA-GVHD and to reduce the side effects of transfusions, transplants, and cancer therapies (Weinmann et al., 2000; Agarwal et al., 2005; Walpurgis et al., 2013; Santacruz-Gomez et al., 2014; Tabatabaei et al., 2016; Acosta-Elías et al., 2017). The blood can be irradiated before, during, and after storage, depending on the patient’s necessities, but a common characteristic of irradiated blood is that RBCs are prone to hemolysis and, therefore, they are more fragile in circulation. This effect reduces the time significantly to storage and transfusion. The Guidelines of the AABB (United States) and the Canadian Standards Association establish that blood can be stored up to 42 days without irradiation and, once irradiated, up to 28 days. In contrast, the Protocols of the Council of Europe and the British Committee for Standards in Haematology allow blood to be stored for up to 28 days, and when blood undergoes the irradiation process, it should be stored for 14 additional days (Góes et al., 2008; de Korte et al., 2018). In México, the current regulation is the Official Mexican Standard NOM-253-SSA1-2012. This standard regulates the disposal of human blood and its components for therapeutic purposes. The storage of unirradiated blood is permitted for up to 42 days after their extraction, in agreement with the United States and Canada regulations. Once the blood is subject to irradiation, the storage time reduces to 14 days.

One approach to assure the quality of irradiated blood is to seek parameters closely related to its primary function: oxygen transportation. Thus, the oxygenation state of the Hb molecule is considered as a reliable parameter to evaluate the quality of both stored and irradiated RBCs (Weinmann et al., 2000; Kanias and Acker, 2010; Acosta-Elías et al., 2015; Mustafa et al., 2016; Acosta-Elías et al., 2017). To discriminate changes that could affect the performance of RBC, it is necessary to take into account changes during storage, e.g., the conversion of erythrocytes into acanthocytes: type 1, with a small central pale halo, and type 2, with a flat surface. Alterations in these cells’ central depth seem to be associated with the presence/absence of oxygen at their surface. Usually, oxygen molecules bind to Hb’s available heme group, forming oxygenated Hb (Oxy-Hb). The presence of oxygen at the surface gives RBC’s a pale central appearance (Santacruz-Gomez et al., 2014). An investigation of Hb functionality revealed that Hb’s oxygenation status is closely related to the ν^4^, νm^37^, and ν^10^ bands’ position and intensity in the Raman spectra. Any modification in these bands depends mostly on the symmetry of the porphyrin ring, revealing whether there have been changes from the tense state (Oxy) to the relaxed state (DeOxy) of Hb (Kanias and Acker, 2010; Acosta-Elías et al., 2017). But it has been found that irradiation and storage do not produce changes in Hb’s oxygenation state until day 13, at doses of radiation ranging from 15 to 50 Gy. However, this does not exclude the possibility that damages in the RBC membrane may effectively interrupt oxygen transport (Acosta-Elías et al., 2015; D’Alessandro et al., 2015). In this respect, the AFM studies may close the gap associated with structural changes in morphology and size of RBC when subjected to radiation and storage stressors.

Studies with AFM have shown dose-dependent damage on mice RBCs after gamma irradiation, with the appearance of nanopores, crater-like vesicles, and changes in membrane roughness (Walpurgis et al., 2013; AlZahrani and Al-Sewaidan, 2017; Taqi et al., 2019). Here, AFM renders invaluable information at nanoscale dominions on RBCs under irradiation stress. Therefore, vesiculation evaluated by AFM is a useful marker for cell aging at the nanoscale (Girasole et al., 2007, 2010). Moreover, the AFM-Raman concurrence permits the evaluation of the oxygenation states in erythrocytes in their different morphologies, such as echinocytes and stomatocytes, to help elucidate whether a rearrangement of the membrane affects oxygen transport (Santacruz-Gomez et al., 2016).

**Figure 1:**
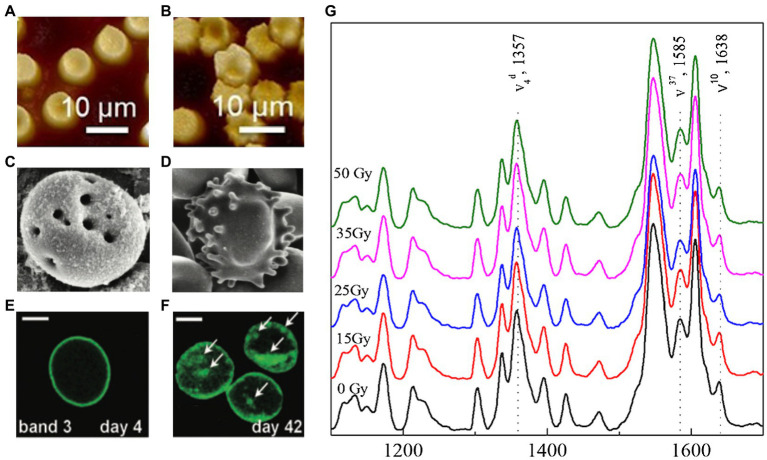
Changes in red blood cells (RBCs) during storage and irradiation. Atomic force microscopy images showing the nanostructure of RBC membrane for **(A)** non-irradiated erythrocytes with homogeneous distribution in shape and size, and **(B)** irradiated erythrocytes with echinocytic morphologies and heterogeneous size distributions. The effects of storage revealed by scanning electron microscopy micrographs of erythrocytes stored after 28 days: **(C)** showing early signs of hemolysis and tiny pores in the membrane and **(D)** RBC with echinocytic morphology. The biochemical modifications induced by storage are visible in the fluorescence micrograph: **(E)** showing the band 3 distribution at day 4, and **(F)** the protein modifications associated with band 3 at day 42. The state of Hb’s oxygenation in RBC subjected to gamma radiation and stored can be tracked with Raman spectra. **(G)** Raman spectrum of Hb after 13 days of storage and at different doses of gamma radiation (15, 25, 35, and 50 Gy). In both cases, no appreciable changes related to oxygen in the heme group configuration are detected. Figures were modified from **(A-B)** Santacruz-Gomez et al. (2016), **(C-D)** Mustafa et al. (2016), **(E-F)** Antonelou et al. (2010), and **(G)** Acosta-Elías et al. (2017).

Figure 1 depicts an illustrative summary of the changes that occur during storage and irradiation. In A and B AFM images, we observed non-irradiated erythrocytes with homogeneous distribution in shape and size. Under gamma irradiation, it is noticed the appearance of echinocytic morphologies and heterogeneous size distributions. SEM micrographs (C and D) show signs of hemolysis with tiny pores in the membrane and echinocytic morphologies after 28 days of storage. Through fluorescence micrographs (E and F), we can observe the protein modifications associated with band 3, highlighting the formation of clusters after prolonged storage of 42 days. Furthermore, with the help of Raman (G) spectroscopy, we know the oxygenation status of Hb after 13 days of storage for different doses of gamma radiation (15, 25, 35, and 50 Gy). Raman spectra showed that erythrocytes subjected to these conditions do not produce changes in Hb’s oxygenation state.

## Implications of RBC Membrane Changes

The main issues in using both irradiated and stored blood are closely related to changes in the biomechanical fingerprint of RBC. This fact impacts the structural quality of RBC for a transfusion and the shelf life of treated blood. The impaired function of RBC also is a serious concern that deserves more profound research. Recently, Acosta-Elías et al. (2017) showed that both storage time and radiation modify one of the most important mechanical properties, the cell surface roughness. In non-irradiated erythrocytes, storage from 0 to 13 days promotes a drastic decrease in the roughness from 2.1 to 0.82 nm. This loss of roughness is related to stiffness and then to the rupture susceptibility of the RBC membrane. Under irradiation, this phenomenon is more evident. For a dose around 50 Gy, RBC membrane roughness values fall among 1.1–0.73 nm. The distribution of the biomechanical response over the membrane associated with the change of elastic properties permits the discrimination of healthy RBC. In this case, RBC is stiffer in its center and softer at the cell periphery, as demonstrated by Ciasca et al. (2015). Another factor that plays a determining role in cell surface nanomechanics is the generation of nanovesicles. The biochemical change promotes the transformation of RBC to spherocytes. These cells have an altered lipid composition that makes the RBC stiffer (Dumitru et al., 2018).

In the medical praxis, nanovesicle release can contribute to posttransfusion effects by stimulating an inflammatory response from the body and causing the blood to coagulate due to the externalized phosphatidylserine (Lentz, 2003; Bosman et al., 2005). The hemoglobin within these vesicles can reduce nitric oxide (NO) availability in the recipient by binding to it, leading to the loss of the vasoregulatory function of NO in the vessels. Reduced NO in the recipient can result in impaired endothelial function, platelet aggregation, and oxidative damage. Prolonged blood storage causes potassium accumulation in the supernatant, linked to cardiac arrhythmia in patients. This excess of potassium means that a particular recipient, such as pediatric patients or heart bypass surgery, cannot be transfused without first removing the excess of potassium (Donadee et al., 2011; Kanias and Gladwin, 2012; Alaarg et al., 2013; Mustafa et al., 2016).

In summary, the quality of blood may be hampered by nanoscales changes on the RBC membrane, reducing transfusion’s effectivity. The required assessment of all biochemical parameters can be accomplished with techniques involving the traditional clinical analysis and nanomechanical-chemical microscopy.

## Discussion and Remarks

The study of the complex biochemical changes on RBC triggered by storing and irradiation stresses may be elucidated by using a set of micro- and nanomicroscopy techniques. In particular, the combination (sequential or simultaneous) of Raman spectroscopy and AFM imaging from the same area in irradiated RBC is a promising alternative. This composed technique may permit the measurement of nanostructured cellular changes and their relation to the conformation of the molecule structure. Hence, generating information on biochemical phenomena affecting the integrity and function of blood cell components is of particular relevance to public health today and remains an active area of research.

## Author Contributions

AL-C, AA-M, AG-E, ES-C, MM-R, and MP-M drafted the work and revised critically for important intellectual content, wrote the paper, and performed the final review of the manuscript. KS-G, MA-E, BC-M, DS-P, OA-B, and AB-E contributed to the conception of the work and performed the final review of the manuscript. All authors contributed to the article and approved the submitted version.

### Conflict of Interest

The authors declare that the research was conducted in the absence of any commercial or financial relationships that could be construed as a potential conflict of interest.
